# Dyslipidemia and Blood-Brain Barrier Integrity in Alzheimer's Disease

**DOI:** 10.1155/2012/184042

**Published:** 2012-05-13

**Authors:** Gene L. Bowman, Jeffrey A. Kaye, Joseph F. Quinn

**Affiliations:** ^1^Department of Neurology, Oregon Health and Science University, 3181 Southwest Samuel Jackson Park Road, Portland, OR 97239, USA; ^2^Biomedical Engineering, Oregon Health and Science University, Portland, OR 97239, USA; ^3^The Portland Veteran Affairs Medical Center, Portland, OR, USA

## Abstract

*Background*. Blood-brain barrier (BBB) dysfunction may have a significant role in the pathogenesis of Alzheimer's disease (AD). Modifiable factors associated with BBB function may have therapeutic implication. This study tested the hypothesis that dyslipidemia is associated with BBB impairment in mild-to-moderate AD. *Methods*. Thirty-six subjects with AD were followed for 1 year. Fasting CSF and plasma were collected with clinical assessments at baseline and 12 months. BBB impairment was defined as CSF albumin index ≥9. Independent *t*-tests and linear regression assessed the relationship between plasma lipoproteins and BBB integrity. *Results*. Dyslipidemia was prevalent in 47% of the population, and in 75% of those with BBB impairment. Subjects with BBB impairment had significantly higher mean plasma triglyceride and lower HDL cholesterol (TG, *P* = 0.007; HDL, *P* = 0.043). Plasma triglycerides explained 22% of the variance in BBB integrity and remained significant after controlling for age, gender, ApoE-4 genotype, blood pressure, and statin use. *Conclusion*. Dyslipidemia is more prevalent in AD subjects with BBB impairment. Plasma triglyceride and HDL cholesterol may have a role in maintaining BBB integrity in mild-to-moderate Alzheimer's disease.

## 1. Introduction

The CSF albumin index is an established measure of blood-brain barrier (BBB) integrity in living patients [1]. This index has detected a higher prevalence of BBB impairment in late onset dementia, including both Alzheimer's disease (AD) and vascular dementia compared to cognitively intact elders [2]. BBB impairment is associated with more rapid rate of decline in AD over 1 year [3]. Capillary endothelia dysfunction and the approximate tight junctions between these cells may be important to AD pathogenesis, including effects on maintaining cerebral perfusion and the clearance of toxic forms of beta-amyloid protein [4, 5]. The notion that the BBB is central to the pathogenesis of AD is controversial, but the enormity of the cerebrovascular tree and the similarity in risk factors between vascular and Alzheimer's disease makes the BBB and neurovascular unit difficult to disregard [6, 7].

If BBB function plays a role in the pathogenesis of AD, then modifiable factors associated with it may have therapeutic potential. A clinical trial of B vitamin supplementation has suggested that the BBB may be a modifiable entity in subjects with hyperhomocysteinemia and mild cognitive impairment [8]. Lipids are plausible candidates for modifying BBB function because of their relationship with vascular disease and ability to affect AD type pathology [9]. This study examines the relationship between dyslipidemia and BBB integrity in a well-characterized sample with mild-to-moderate Alzheimer's disease.

## 2. Methods

### 2.1. Study Population

The study participants were recruited from the clinic population of the NIA—Layton Aging and Alzheimer's Disease Center of Oregon Health and Science University and have been described previously [3]. Briefly, all subjects had a consensus diagnosis of probable AD using the National Institute of Neurological and Communicative Disorders and Stroke/Alzheimer's Disease and Related Disorders Association criteria and a Clinical Dementia Rating of 0.5 or 1 to establish disease stage of mild-to-moderate AD. All participants provided informed consent in accord with the Institutional Review Board for human study at Oregon Health and Science University. All subjects had CSF and blood available for analysis. Twenty-three of these 36 subjects have come to brain autopsy, and in all autopsy cases the clinical diagnosis of AD was confirmed pathologically.

### 2.2. Data Collection and Analysis

Thirty-six subjects with mild-to-moderate AD were evaluated by medical history, neurological and general examination, the Mini-Mental State Examination (MMSE) [10], the Clinical Dementia Rating scale (CDR) [11], the modified Hachinski ischemia score [12], and the Geriatric Depression Scale [13]. Cerebrospinal fluid and peripheral blood were collected, and brain MRI was performed.

Lumbar punctures were performed in the morning under standardized conditions at L3 to L4 or L4 to L5 interspaces, immediately aliquoted, and snap frozen at −70°C until assayed. These samples had normal cell count and glucose levels, and the aliquots analyzed were matched sequentially by draw to control for CSF content drift by sequence. A CSF-to-serum ratio of albumin (CSF Albumin Index) ≥ 9.0 was considered BBB impairment. Reproducibility of the CSF albumin index in AD over 1-year has been established (intraclass correlation coefficient =  .96) [3]. Blood samples collected at the same visit as CSF were analyzed for albumin, glucose, triglycerides, total cholesterol, and high- and low-density lipoproteins by standardized methods performed by the Atherosclerosis Lipid Research Laboratory at Oregon Health and Science University [14]. Laboratory staff was blind to all clinical covariates.

### 2.3. Statistical Analysis

#### 2.3.1. Descriptive Analysis

Two sample *t-*tests compared the mean differences in CSF albumin Index by subjects classified as atherogenic dyslipidemia (triglycerides ≥ 150 mg/dL, HDL cholesterol < 50 mg/dL and LDL cholesterol > 100 mg/dL [15]) and metabolic dyslipidemia (the atherogenic profile without reference to LDL cholesterol) [16]. Mean differences in each lipid were compared between subjects with and without BBB impairment.

#### 2.3.2. Primary Analysis

Multivariable linear regression models were fit to assess the relationship between lipids and CSF albumin index by including potential confounders including age, gender, ApoE-4 genotype, blood pressure, and statin use. Alpha level for significance was set at 0.05 (2-tailed).

## 3. Results

**Figure 1:**
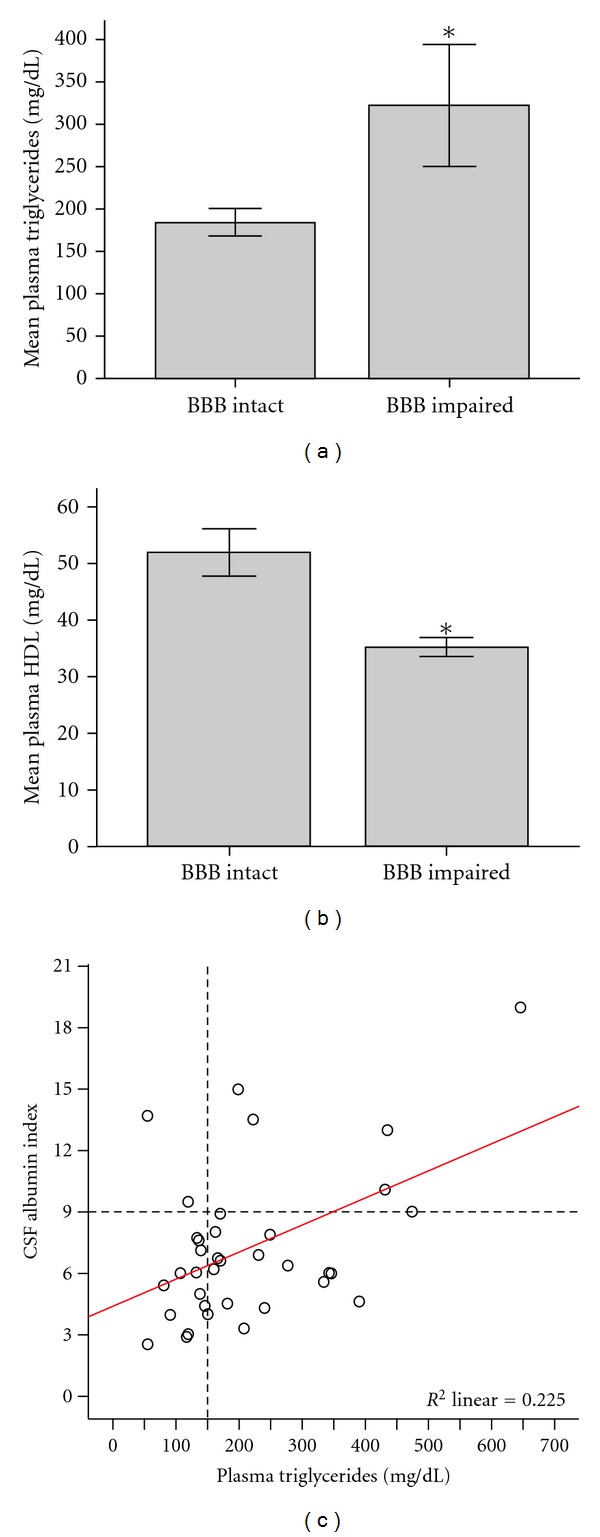
Relationship between plasma lipids and blood-brain barrier in subjects with AD. (a) Mean difference in TG = 137.89 mg/dL, *P* = 0.007. (b) Mean difference in HDL = 16.17 mg/dL, *P* = 0.043. Standard error bars around the mean set at +/−1.0. (c) Horizontal reference line separates the plots by subjects with (above) and without (below) BBB impairment. Vertical reference line indicates lipid risk threshold.

Table 1 summarizes the study population. Blood-brain barrier (BBB) impairment was prevalent in 22%. Frequency of statin use was similar between the groups with and without BBB impairment (*P* for difference = 0.251). Total and LDL cholesterol were not different between the two groups (total cholesterol, *P* = .619; LDL cholesterol, *P* = .355). However, mean plasma triglycerides were higher and HDL cholesterol lower in subjects with BBB impairment (mean difference in TG = 137.89 mg/dL, *P* = 0.007; mean difference in HDL = 16.17 mg/dL, *P* = 0.043) (Figures 1(a) and 1(b)). Seventy-five percent (6/8) with BBB impairment had plasma triglycerides ≥150 mg/dL (range 55–646 mg/dL) compared to 57% (16/28) in subjects with intact BBB (range 55–389 mg/dL). Everyone with BBB impairment had plasma HDL cholesterol below 50 mg/dL (range 29–43) compared to 60% (17/28) in subjects with intact BBB (range 23–106 mg/dL).

### 3.1. Dyslipidemia and BBB Impairment in Alzheimer's Disease

The overall prevalence of “atherogenic” and “metabolic” dyslipidemia was 42% and 47%, respectively. The prevalence of atherogenic dyslipidemia was more frequent in AD subjects with BBB impairment than in subjects without (75% versus 32% with intact BBB, *P* = 0.030, Table 1). Metabolic dyslipidemia was also more prevalent in BBB impaired (75% versus 39% with intact BBB, *P* = 0.048). Subjects with atherogenic dyslipidemia (*n* = 15) had mean CSF Albumin Index 2.69 units higher than those without (*n* = 21) (*P* = .029). Subjects with metabolic dyslipidemia (*n* = 17) had mean CSF Albumin Index 2.43 units higher than those without (*n* = 19) (*P* = 0.48).

### 3.2. Lipids Associated with BBB Integrity in Alzheimer's Disease

Linear regression analysis indicated that each 10 mg/dL increase in plasma triglyceride content was associated with a 0.13 unit increase in CSF Albumin Index (*P* = 0.004) (Figure 1(c)). Plasma triglycerides explained 22.5% of the variation in BBB integrity. The association between plasma triglycerides and CSF Albumin Index remained significant in multivariable regression analysis controlled for age, gender, ApoE-4 carrier status, systolic blood pressure, and statin use (*P* = 0.040). Total cholesterol, LDL cholesterol, and HDL cholesterol were not associated with CSF Albumin Index in the regression model (data not shown).

## 4. Conclusion

These findings demonstrate a higher prevalence of dyslipidemia in Alzheimer's subjects with BBB impairment. Plasma triglycerides and HDL cholesterol were the lipids most associated with BBB integrity. Plasma triglycerides explain the most variation in BBB integrity compared to HDL, LDL, and total cholesterol.

A relationship between triglycerides and dementia has been reported in one large study [17]. This group compared the various lipid components contributing to dementia risk but did not report data on BBB integrity. To our knowledge, this is the first study to report a significant relationship between dyslipidemia and BBB integrity in Alzheimer's disease. While a causal relationship between plasma lipids and BBB cannot be assumed, the most plausible explanation may be that BBB integrity in AD detected by the CSF Albumin Index is related to atherosclerosis and its risk factors. For example, the omega 3 polyunsaturated fatty acids, eicosapentaenoic acid, and docosahexaenoic acid are associated with both vascular disease and dementia risk and are known to lower plasma triglyceride levels [18]. It is also plausible that the effect of dyslipidemia on the BBB is independent of atherosclerosis. This has been observed in the case of an inborn error of cholesterol metabolism with neurologic complications, cerebrotendinous xanthomatosis [19].

Although sample size in this study is a limitation, we believe that the study has particular strengths worth noting. Sixty four percent of this study population has autopsy confirmed AD, and the entire sample had probable AD confirmed by consensus diagnosis. Another strength is the stability of the CSF Albumin Index as a measure of BBB integrity in living subjects [3] and the collection of fasting blood for the analysis of plasma lipids. These attributes minimize both risk of exposure and outcome misclassification and therefore strengthen the validity of the study findings in a limited sample.

The possibility that dyslipidemia is causally related to BBB impairment may be clinically significant since dyslipidemia is treatable. If this evidence is confirmed in other populations, then the next step may involve a lipid-modifying strategy to modify the natural history of AD. While it is true that statin therapy has been unsuccessful in altering the course of AD, these current findings place emphasis on modifying triglyceride and HDL cholesterol, ideally in subjects selected on the basis of BBB impairment at baseline. Perhaps a dietary pattern or supplementation with omega-3 PUFAs and niacin would offer one strategy, since they favorably modify triglyceride and HDL cholesterol metabolism, respectively [20]. The emergence of imaging modalities for the assessment of BBB integrity will make these types of intervention more feasible.

## Figures and Tables

**Table 1:** Study population characteristics^1^.

	Total (*n* = 36)	BBB intact (*n* = 28)	BBB impaired (*n* = 8)
Age, years	70 (7.1)	70 (7)	73 (8)
Female, no. (%)	12 (33)	11 (39)	1 (13)
ApoE*-e*4 allele carriers (%)	27 (75)	21 (75)	6 (75)
BMI (%)	27 (4.6)	26 (4)	29 (6)
BP, systole, mmHg	142 (23.2)	143 (21)	137 (30)
BP, diastole, mmHg	78 (12.2)	79 (13)	76 (8)
Mini mental state exam	19 (5.0)	20 (5)	18 (5)
Hachinski ischemia score	0.6 (0.9)	0.5 (0.8)	1.0 (1.3)
Statin use, no. (%)	8 (22)	5 (18)	3 (38)
Glucose, mg/dL	99 (18)	98 (18)	101 (17)
CSF albumin index	7.2 (3.7)	5.6 (1.7)	12.9 (3.3)^abc^
Atherogenic dyslipidemia, no. (%)^2^	15 (42)	9 (32)	6 (75)^a^
Metabolic dyslipidemia, no. (%)^2^	17 (47)	11 (39)	6 (75)^a^
Triglycerides, mg/dL	215 (132)	185 (86)	323 (204)^ab^
HDL cholesterol, mg/dL	48 (20)	52 (22)	35 (5)^a^
LDL cholesterol, mg/dL	129 (33)	132 (37)	120 (10)
Total cholesterol, mg/dL	216 (36)	218 (40)	210 (27)

BBB: blood-brain barrier, ApoE-*e*4: apolipoprotein E epsilon 4, BMI: body mass index, BP: blood pressure, HDL: high-density lipoprotein, LDL: low-density lipoprotein.

^1^Mean and standard deviation provided unless stated otherwise; ^a^ *P* < 0.05, ^ab^ *P* < 0.01, ^abc^ *P* < 0.001.

^2^Atherogenic dyslipidemia: triglycerides ≥ 150 mg/dL, LDL > 100 mg/dL and HDL < 50 mg/dL [15].

^3^Metabolic dyslipidemia: triglycerides ≥ 150 mg/dL, HDL < 50 mg/dL [16].
